# Endoscopic diagnosis and treatment in gastric cancer: Current evidence and new perspectives

**DOI:** 10.3389/fsurg.2023.1122454

**Published:** 2023-04-04

**Authors:** Áron Vincze

**Affiliations:** Division of Interventional Gastroenterology and Division of Gastroenterology, First Department of Medicine, Faculty of Medicine, University of Pécs, Pécs, Hungary

**Keywords:** early gastric cancer, gastric premalignant lesions, missed gastric cancer, optical characterization, quality parameters of upper gastrointestinal endoscopy, endoscopic submucosal dissection

## Abstract

Gastric cancer is the fifth most common cause of cancer related deaths worldwide. Despite advancement in endoscopic techniques, the majority of the cases are diagnosed at late stage, when the curative treatment options are very limited. The early gastric cancer (EGC) on the other side is potentially curable, and in selected cases endoscopic resection techniques offer similar survival rates then surgical resection. The detection of EGC is endoscopically challenging and requires high quality examination. Recent data show that close to 10% of the gastric cancer cases had a previous negative endoscopy. This highlights the urgent need to improve the quality of the endoscopy services, what can be achieved by increasing the awareness of gastroenterologists and continuously monitoring the key performance indicators of upper gastrointestinal endoscopy. Newer endoscopic imaging techniques are also becoming commonly available to aid the detection of gastric premalignant lesions and EGC. High-definition endoscopy with image enhancement techniques is preferred over white light endoscopy to recognize these lesions, and they are also useful to determine the invasion depth of EGC. The endoscopic optical characterization of lesions is necessary for the selection of proper resection method and decide whether endoscopic resection techniques can be considered. Artificial intelligence systems aid the detection of EGC and can help to determine the depth of invasion. Endoscopic mucosal resection and endoscopic submucosal dissection requires centralized care and tertiary referral centers with appropriate expertise to ensure proper patient selection, high success rate and low adverse event rate. Appropriately scheduled endoscopic surveillance of high-risk patients, premalignant lesions and after resection of EGC is also important in the early detection and successful treatment of gastric cancer.

## 1. Introduction

Gastric cancer is the fifth most common cause of cancer related deaths worldwide (1). Despite advancement in endoscopic techniques, large proportion of the cases are diagnosed at late stage, when the curative treatment options are very limited. Early gastric cancer (EGC) on the other side is potentially curable, and in selected patient's endoscopic resection techniques offer similar survival rates then surgical resection. Early detection of gastric cancer can significantly improve the expected survival rate. The 5-year survival rate of GC patients in most countries is approximately 20%, whereas that of early GC (EGC) can reach 90% (2). Upper gastrointestinal (UGI) endoscopy is the gold standard to diagnose gastric cancer. The detection of EGC is endoscopically challenging and requires high quality examination.

## 2. Endoscopic diagnosis and treatment of gastric cancer

### 2.1. Missed gastric cancer

Several studies indicate that similarly to colorectal cancer, a significant proportion of gastric cancers are so called interval cancers, which were missed during an earlier upper GI endoscopy. The time frame between the earlier endoscopy and the endoscopy which detected the cancer was usually within 3 years in most studies. Cancers detected within 1 year after a previous negative endoscopy are considered missed cancers, and those detected between 1 and 3 years are considered possible missed cancer. This is based on the doubling time of 2–3 years for gastric adenocarcinoma (3), which leads to the assumption that a cancer diagnosed within a year after a normal UGI endoscopy would almost certainly have been present as a macroscopic lesion at the time of the initial endoscopy and therefore had been missed.

An earlier meta-analysis of 10 studies, including 3,787 subjects showed that 12.9% of the patients had undergone upper GI endoscopy that missed cancer in the preceding 3 years, and 85% of these were gastric cancers (4). The possible explanations of the missed premalignant or malignant gastric lesions are inadequate supervision of trainees, lack of patient tolerance due to inadequate sedation, inappropriate follow up, errors in mucosal sampling and histopathologic interpretations. A more recent meta-analysis included 22 studies with a significantly higher patient number also explored the missing rate of gastric cancer. The author analyzed the data of 69.061 patients, and concluded that the missed gastric cancer proportion was 9.4% (5). In this study, younger age (<55 years), female sex, marked gastric atrophy, gastric adenoma or ulcer, and inadequate number of biopsy fragments were reported as predictive factors for diagnostic failure.

### 2.2. Quality of endoscopy

The problem of missed gastric cancer highlights the urgent need to improve the quality of the endoscopy services, which can be achieved by increasing the awareness of gastroenterologists and continuously monitoring the key performance indicators of upper gastrointestinal endoscopy. Appropriate patient preparation with administration of defoaming and mucolytic agents before/during endoscopy (6), and adequate inspection of the gastric mucosal surface are some of the main determinants of high-quality examination. Adequate inspection can be ensured with appropriate insufflation to flatten the mucosal folds, systematic inspection, and photo documentation.

To improve the detection of pathology, similarly to colonoscopy withdrawal time, it is also suggested to measure the duration of the examination. One of the key performance indicators of upper GI endoscopy in the recent ESGE Quality Improvement Initiatives is that the entire procedure should last minimum 7 min from scope intubation to extubation (7). This is based on the study of Teh et al., who showed that “slow endoscopist” (mean duration of endoscopy was 8.6 ± 4.2 min) was three times more likely to detect a neoplastic lesion (cancer and dysplasia alone) in the stomach compared to a fast endoscopist (mean duration of endoscopy was 5.5 ± 2.1 min, OR 3.42, 95% CI 1.25–10.38) (8). Another important key performance indicator is the accurate photodocumentation, which is believed to improve the quality of endoscopy, however no data supports this so far (7). Nowadays accurate and good quality image documentation is recommended by major endoscopic societies, and not only the abnormal findings, but the normal landmarks are also expected to be captured and incorporated to the hospital information systems (7, 9).

### 2.3. Optical characterization of gastric lesions

Appropriate endoscopic identification and characterization of suspicious lesions in the stomach is very important. The endoscopic optical characterization of lesions is necessary for the selection of proper resection method and decide whether endoscopic resection techniques can be considered. Focal erythema or whitish discoloration, irregular mucosal surface with protrusions, elevations or depressions, spontaneous bleeding and abnormal mucosal folds are the most important hallmarks of early gastric cancer.

These lesions should also be characterized by the Paris classification (10) as polypoid (type I), flat (type II) or excavated (type III) lesions. Flat, type II lesions also might have some elevation which is less than 1.3 mm (IIa), or they might be completely flat (IIb) or superficially depressed (IIc). Flat, depressed, or excavated lesions have significant higher chance of submucosal invasion, which influence the endoscopic resecability.

Further signs were also analyzed to determine the depth of invasion with conventional endoscopy. The non-extension sign is seen when the gastric wall is distended by insufflation, but a trapezoid elevation remains visible at the site of early gastric cancer. This indicates that the cancer is causing a deeper infiltration of the submucosa (500 μm or more), which is labeled as SM2. The specificity and sensitivity of the non-extension sign was 97.7% and 92.0% in a large cohort (11). These SM2 lesions are not suitable for endoscopic resection since the risk of lymph node metastasis is significant at this stage.

Newer endoscopic imaging techniques are also becoming commonly available to aid the detection of gastric premalignant lesions and EGC. High-definition endoscopy with image enhancement techniques is preferred over white light endoscopy to recognize these lesions, and they are also useful to determine the invasion depth of EGC. Image enhancement techniques or advanced endoscopic imaging are the narrow band imaging (NBI, Olympus), Fuji intelligent chromoendoscopy (FICE, Fujifilm), blue laser imaging (BLI, Fujifilm), linked color imaging (LCI, Fujifilm), i-scan with surface enhancement, contrast enhancement and tone enhancement modes (Pentax). These advanced techniques are based on specific blue and green wavelengths to enhance the mucosal surface pattern and the mucosal/submucosal microvessels. These wavelengths correspond to the light absorption of hemoglobin, which result more contrast and better visualization of capillaries in the endoscopic image. These techniques increase the detection of early neoplastic lesions in the colorectal area and in the esophagus according to large number of studies, but less information is available for EGC. The usefulness of advanced imaging in the detection of EGC is still under discussion (6). High-definition white light endoscopy and NBI had similar detection rate of gastric cancer, but NBI performed better in the identification of intestinal metaplasia in a multicenter randomized controlled study (12). Data from another multicenter randomized controlled study show high rate of accuracy and specificity (>90%), but lower rate of sensitivity (60%) for depressed small (<1 cm) gastric cancer with magnifying NBI, and these values were significantly better than those of white-light endoscopy (13).

The Japanese Society of Gastroenterology and Japanese Gastric Cancer Association jointly advocate the magnifying endoscopy simple diagnostic algorithm for gastric cancer based on the vessel and surface (VS) classification system. A lesion with demarcation line between cancerous and normal mucosa plus irregular microvascular or surface pattern is highly suspicious for EGC (14). Demarcation line is the border of the lesion, where an abrupt change can be observed in the microvascular and microsurface pattern. Image-enhanced magnifying endoscopy is particularly useful in the diagnosis of differentiated-type early gastric cancer. Beside the VS classification system, other characteristic findings of EGC during magnifying endoscopy are also described in a recent review. These are the presence of white opaque substance, light blue crest, white globe appearance, vessel within epithelial circle pattern (15).

### 2.4. Artificial intelligence in the detection of early gastric cancer

Several studies have been published about the role and future perspectives of artificial intelligence (AI) in the detection of EGC, which were recently reviewed by Xiao Z et al. (16). Among these different systems, real time assistance is also available to ensure that the entire mucosal surface of the stomach is visualized during the endoscopy, what is a prerequisite for the detection of early neoplastic changes. The system was named as WISENSE (from the words of wise and sense) and was compared to the conventional endoscopy in a randomized controlled trial involving 324 patients. The rate of blind spots was significantly less using WISENSE than in the controls (5.9% vs. 22.4%, *p* < 0.001) (17). The Gastrointestinal Artificial Intelligence Diagnostic System (GRAIDS) is another real time tool developed by using more than 1 million endoscopic images taken from more than 80.000 patients. The system can detect upper GI cancer with sensitivity similar to that of expert endoscopist and superior to that of non-expert endoscopist in real time (18).

The invasion depth can be also evaluated by using convolutional neural network computer-aided detection, and it was shown that accuracy and specificity of this AI method is significantly better than that of experienced endoscopists (19). ENDOANGEL is also a real-time AI system that covers various aspects of EGC diagnosis, including detection with white light endoscopy, magnifying narrow band imaging, and predicting invasion depth (2). The speciﬁcity, accuracy, and positive predictive value of ENDOANGEL (93.22%, 91%, and 90%, respectively) were signiﬁcantly higher than those of endoscopists (72.33%, 76.19%, and 70.56%, respectively), and its sensitivity and negative predictive value were slightly higher than those of endoscopists (2).

### 2.5. Endoscopic resection techniques

The identified and carefully characterized neoplastic lesions should be resected in an *en bloc* fashion (20, 21). The main endoscopic techniques are endoscopic mucosal resection (EMR) and endoscopic submucosal dissection (ESD), both requires centralized care and tertiary referral centers with appropriate expertise to ensure proper patient selection, high success rate and low adverse event rate. Significantly higher R0 *en bloc* resection rates can be achieved by ESD; therefore, this is the recommended technique, especially for lesions larger than 10–15 mm. EMR is acceptable for smaller lesions with a very low probability of advanced histology. Non-lifting lesions are also not suitable for EMR, because it does not allow proper histological evaluation of EGC. ESD is suggested to be the first line resection technique for gastric neoplasia in cases with very low risk of deep submucosal invasion and lymph node metastasis. ESD in the stomach is recommended for dysplasia or intramucosal carcinomas of any size without ulceration or <30 mm if ulcerated. Well differentiated superficial or SM1 adenocarcinomas of size <30 mm, or poorly differentiated intramucosal adenocarcinomas of size <20 mm without ulceration can be also considered for ESD, but it should be based on individual decision, since the recommendation is weak (21).

ESD is technically demanding, requires significantly longer procedure time and carries higher risk of adverse events, mainly perforations, if compared to EMR (22). ESD offers an alternative for surgical resection for highly selected patients with EGC, since this technique is less expensive, and associated with less perioperative morbidity, faster recovery, shorter length of hospital stay and better quality of life, according to the most comprehensive meta-analysis (23). On the other hand, ESD is related with higher risk of recurrence, metachronous and synchronous cancer compared to surgery, therefore strict and close follow-up is advised after endoscopic removal.

This endoscopic resection technique became widely available in the Far East more than 10 years ago, and many Western endoscopist visited Japan to learn ESD. To further increase the availability of this advanced endoscopic procedure in Europe, ESGE developed a core curriculum for ESD practice to ensure proper training, competence and maintain proficiency in the technique (24).

Depending on the histological features of the resected specimen, curative and noncurative resections should be discriminated, carrying low (<3%) and high risk of lymph node metastasis [Table 1 (21),]. Patients with noncurative, high risk resection should have complete staging after resection and additional surgical treatment should be offered. Further risk assessment of the endoscopic curability can be carried out by the eCura scoring system as it is suggested by the Japanese gastric treatment guidelines (25). The score can discriminate between low (0–1 point), intermediate (2–4 points) and high risk lesions, where lymphatic invasion is 3 points, and 1–1 point is added for tumor size >30 mm, SM2 status, venous invasion and positive vertical margin (26).

**Table 1:** Distinction between curative and noncurative endoscopic resection of early gastric cancers.

Main features	Low risk (curative)	High risk (noncurative)
R0, intramucosal, well to moderately differentiated	– any size without ulceration, or – ≤30 mm with ulceration	– >30 mm with ulceration
R0, SM1, well to moderately differentiated	– ≤30 mm and – no lymphovascular invasion and – no ulcers	– >30 mm or – lymphovascular invasion or – with ulceration
R0, intramucosal, poorly differentiated	– ≤ 20 mm and – no lymphovascular invasion and – no ulcers	– >20 mm or – lymphovascular invasion or – with ulceration

### 2.6. Risk stratification and surveillance

Appropriately scheduled endoscopic surveillance of high-risk patients, premalignant lesions and after resection of EGC is also important in the early detection and successful treatment of gastric cancer.

Helicobacter pylori infection is a well-known promoter of gastric carcinogenesis by inducing chronic inflammation, which leads to atrophic gastritis, intestinal metaplasia, dysplasia and finally intestinal-type gastric adenocarcinoma. Patients with chronic atrophic gastritis and intestinal metaplasia are at risk of gastric adenocarcinoma (27). The more severe and extensive are the atrophy and the intestinal metaplasia, the higher is the risk of early gastric cancers. Therefore, it is very important to correctly determine the stage and extension of these abnormalities, but conventional endoscopic visualization of atrophy and intestinal metaplasia correlates poorly with the histological findings. The best way to estimate the severity and extension of these changes is random biopsy sampling from the stomach, according to the Sydney protocol: 2 samples from the antrum, 2 from the corpus (small and large curvature in both case) and an additional sample from the incisura. The samples are evaluated according to the Operative Link of Gastritis Assessment (OLGA) and Intestinal Metaplasia (OLGIM) strategy (27).

Mild or moderate atrophy localized in the antral area does not require surveillance, while those patients who have severe atrophy or intestinal metaplasia in both antrum and corpus (OLGA/OLGIM III/IV stages) should be followed up with a high quality endoscopy and biopsies in 3 years interval (27). Those patients who have family history of gastric cancer may benefit a more frequent follow-up, e.g., every 1–2 years.

Endoscopic surveillance is advised 3–6 month after curative resection of EGC with high-definition white light and chromoendoscopy, and then annually. Other cross-sectional imaging methods (EUS, CT, MRI, PET) are not advised routinely in these cases (21).

## 3. Conclusions

The detection of EGC is endoscopically challenging and requires high quality examination. Recent data show that close to 10% of the gastric cancer cases had a previous negative endoscopy. This unfavorable phenomenon can be markedly reduced if the quality of endoscopic evaluation is improved. Newer endoscopic imaging techniques are also becoming widely available to help the optical characterization, which can be further enhanced by artificial intelligence. Real time AI systems capable to detect and characterize premalignant/malignant gastric lesions are becoming available in the foreseeable future. Proper endoscopic evaluation of EGC is also required for the adequate selection of resection techniques, since usually cross-sectional imaging methods are not able to identify and characterize these small malignant gastric lesions. ESD offers an alternative for surgical resection for highly selected patients with EGC. Appropriately scheduled endoscopic surveillance of high-risk patients, premalignant lesions and after resection of EGC is also important in the early detection and successful treatment of gastric cancer.
